# From molecular descriptions to cellular functions of intrinsically disordered protein regions

**DOI:** 10.1063/5.0225900

**Published:** 2024-11-25

**Authors:** Wei Chen, Olivia A. Fraser, Christy George, Scott A. Showalter

**Affiliations:** 1 Department of Chemistry, The Pennsylvania State University, University Park, Pennsylvania 16802, USA; 2 Department of Biochemistry and Molecular Biology, The Pennsylvania State University, University Park, Pennsylvania 16802, USA

## Abstract

Molecular descriptions of intrinsically disordered protein regions (IDRs) are fundamental to understanding their cellular functions and regulation. NMR spectroscopy has been a leading tool in characterizing IDRs at the atomic level. In this review, we highlight recent conceptual breakthroughs in the study of IDRs facilitated by NMR and discuss emerging NMR techniques that bridge molecular descriptions to cellular functions. First, we review the assemblies formed by IDRs at various scales, from one-to-one complexes to non-stoichiometric clusters and condensates, discussing how NMR characterizes their structural dynamics and molecular interactions. Next, we explore several unique interaction modes of IDRs that enable regulatory mechanisms such as selective transport and switch-like inhibition. Finally, we highlight recent progress in solid-state NMR and in-cell NMR on IDRs, discussing how these methods allow for atomic characterization of full-length IDR complexes in various phases and cellular environments. This review emphasizes recent conceptual and methodological advancements in IDR studies by NMR and offers future perspectives on bridging the gap between *in vitro* molecular descriptions and the cellular functions of IDRs.

## I. INTRODUCTION

Comprising over 50% of the human proteome,^1^ intrinsically disordered protein regions (IDRs) play a pivotal role in protein–protein interactions that support nearly every aspect of cellular function.^2–4^ IDRs lack defined secondary and tertiary structures,^5^ with lengths ranging from short linkers and tails (5–10 residues) to regions containing over 1000 residues. Each IDR is unique; some become ordered upon binding to partners,^6^ some adopt different conformations when bound to different partners,^7^ while some remain completely disordered in their bound state.^8^ These distinctive properties, encoded in the protein sequence and influenced by the environment,^9^ allow IDRs to respond readily to cellular signals. Understanding these properties and their functional outcomes requires experimental characterization of conformational ensembles and binding interactions.

Nuclear magnetic resonance (NMR) spectroscopy has been a powerful tool in discovering and studying IDRs and remains essential for their biophysical characterization.^10–13^ NMR provides atomic-level insights into structures, motions, thermodynamics, and kinetics of IDRs and their interactions. Over the past few decades, NMR has led to a plethora of quantitative molecular descriptions of IDRs, including their disorder-to-order transitions, specificity and plasticity in binding interactions, and structural dynamics, which have been extensively reviewed.^14–17^

Here, we review several recent key conceptual breakthroughs in IDRs facilitated by NMR, and highlight technical advances for bridging molecular descriptions to cellular functions. We first review the assemblies formed by IDRs at various scales. Recently, a large number of IDRs has been identified to form biomolecular condensates via liquid–liquid phase separation (LLPS). NMR has been applied to characterize various timescales of the dynamics and molecular interactions within these condensates.^18,19^ Here, we focus on the assemblies formed by IDRs at various scales: from one-to-one complexes to non-stoichiometric dynamic clusters and condensates. Next, we discuss the dynamic interactions of IDRs driven by multivalency and conformational flexibility. Accumulation of biophysical characterization of IDRs is beginning to reveal the unique consequences of their structural properties on their functions. Despite the current knowledge gap between biophysical studies and cellular functions of IDRs, several unconventional thermodynamic and kinetic schemes have emerged from *in vitro* characterization of IDR interactions,^20–26^ which can already imply regulatory mechanisms in the cell. Here, we discuss these thermodynamic and kinetic schemes, driven by the combination of multivalent interactions and conformational flexibility of IDRs, and their functional implications.

Moving toward a more comprehensive understanding of the cellular behaviors of IDRs from *in vitro* molecular descriptions, it becomes necessary to look at IDRs in the context of full-length proteins and large multicomponent complexes, where folded domains are also present. This is particularly challenging for solution NMR because globular proteins with sizes larger than ∼25 kD are difficult to detect due to their rapid transverse relaxation.^27^ Furthermore, since the conformational ensemble of IDRs are highly sensitive to environmental changes, it is also crucial to be able to study them in the cellular context. Here, we highlight emerging NMR methods for tackling large proteins in various phases and cellular environments and how these methods can contribute to bridging molecular description of IDRs to their cellular functions.

## II. ASSEMBLIES OF INTRINSICALLY DISORDERED PROTEINS AT VARIOUS SCALES

**FIG. 1.**
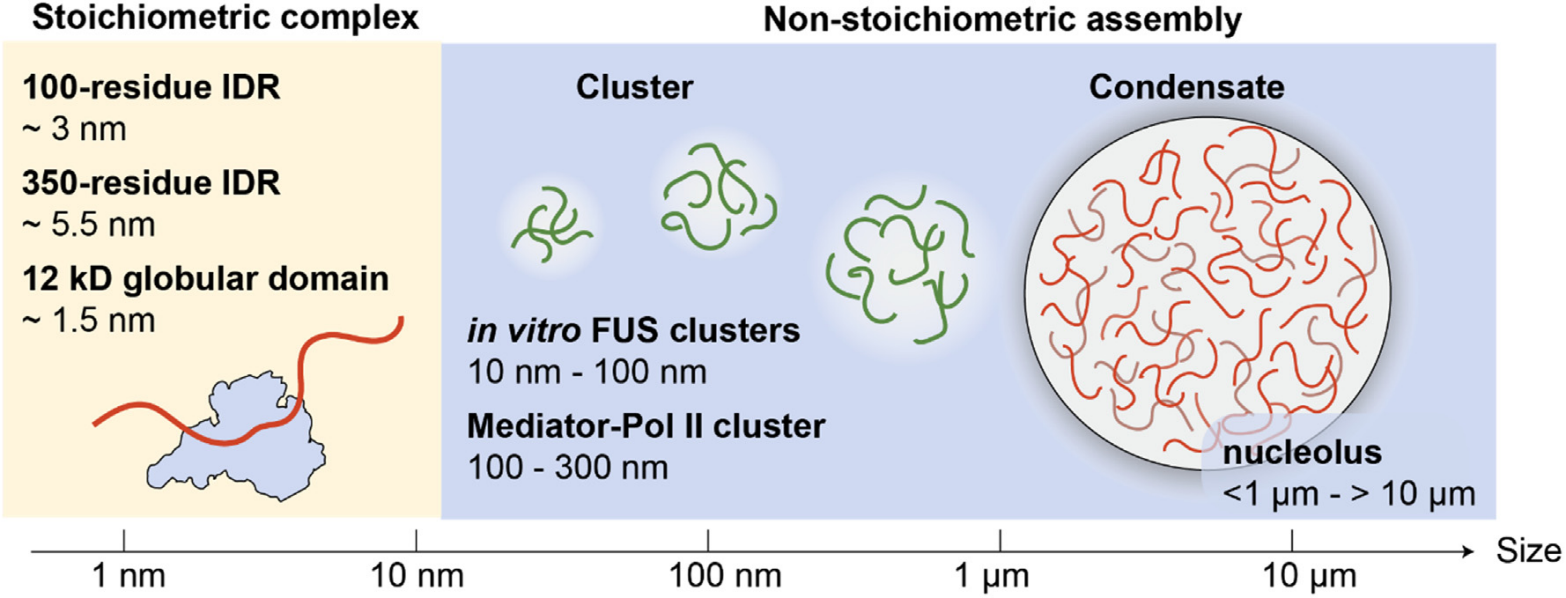
Assemblies of intrinsically disordered regions (IDRs) at various scales. IDRs form stoichiometric complexes with defined molar ratios. For example, an IDR with ∼100 residues has a radius of gyration (R_g_) of approximately 3 nm,^29^ an IDR with ∼350 residues has an R_g_ of approximately 5.5 nm,^30^ and a 12 kD globular protein domain has an R_g_ of approximately 1.5 nm.^31^ IDRs also form non-stoichiometric assemblies, where the protein molar ratio is not fixed. For example, below the saturation concentration for liquid-liquid phase separation (LLPS), FUS proteins form clusters ranging from 10 to 100 nm in size,^32^ and Mediator-RNA clusters in cells range from 100 to 300 nm.^33^ IDR condensates formed through LLPS are larger, with membraneless organelles such as the nucleoli ranging in size from less than 1 *μ*m to over 10 *μ*m.^34^

IDRs participate in biomolecular interactions in many ways. While they can form well-defined protein complexes with fixed molar ratios (stoichiometric), an increasing number of studies show that IDRs also form assemblies of which the molar ratio of participating proteins is not well defined (non-stoichiometric)^28^ (Fig. 1). Here, we discuss these assemblies of IDRs at various scales and recent NMR efforts to characterize their dynamics and molecular interactions.

### A. Stoichiometric complexes

The major focus of IDR interactions has been on stoichiometric complexes, also known as discrete complexes,^20^ where the molar ratio of each protein component in the complex is well defined. Identifying binding sites at the residue level with NMR has been a common practice and particularly useful when the interactions are too weak or dynamic for x-ray crystallography to capture. Techniques like chemical shift titration and ^15^N transverse relaxation rate (R_2_) measurement are commonly used to identify residues affected by binding, reflected in changes in positions and/or intensities of the backbone chemical shift and increased R_2_. However, complex formation can often lead to line broadening for IDRs, caused by the large size of the complex formed or microsecond to millisecond conformational exchange, resulting in signal loss. Although such lack of direct indication of binding may not be ideal, the microsecond to millisecond exchange can be exploited for further NMR analyses such as chemical exchange saturation transfer (CEST) and Carr–Purcell–Meiboom–Gill (CPMG) relaxation dispersion experiments to characterize the populations, kinetics, and chemical shifts of the exchanging states.^35^

Beyond identifying residues affected by binding, NMR is powerful for characterization of structure and dynamics at the binding interface. For example, using transferred-NOE, specific methyl labeling, and ^13^C-edited/^13^C-filttered NOESY experiments, Anglister and coworkers elucidated pair-wise contacts between the STEP kinase interactions motif and the protein p38α,^36^ providing a distinction for residues exhibiting chemical shift perturbation due to direct contacts or long-range allosteric communications. NMR can also quantify dynamic interactions, such as the conformational exchange associated with the binding of the intrinsically disordered regulatory region of MKK4 to p38α, which Jensen and coworkers characterized using a combination of rotating-frame R1ρ, CPMG relaxation dispersion, and CEST.^37^ Their results show that MKK4 and p38α form a partially fuzzy complex where MKK4 uses its docking site motif as a rigid anchor while its KIS domain undergoes rapid dynamics while remaining on the surface of p38α.

### B. Biomolecular condensates

An increasing body of cellular work has revealed biomolecular assembly with the size of hundreds of nanometers to micrometers in both the nucleus and the cytoplasm.^33,38–40^ IDRs have been identified to play an important role in these assemblies, although they are not necessary in every system. The diffusion of proteins in and out of the assembly measured by fluorescence recovery after photobleaching (FRAP) shows rapid exchange of molecules between the assembly and the surrounding,^40,41^ highlighting the dynamic nature. These assemblies have also been shown to have liquid-like behaviors such as merging, and therefore, are often referred to as droplets or condensates.^39,40^ The current working model for biomolecular condensate formation is via the mechanism of liquid–liquid phase separation (LLPS).^42^ In the LLPS model, beyond a critical concentration, proteins separate into two distinct phases—a dispersed phase where protein concentration remains low and a condensed phase where proteins are concentrated within the droplets or condensates. Given the prevalence of biomolecular condensates observed in cells, it becomes of great interest to understand the behaviors of proteins in the condensed phase and the driving force behind LLPS.

NMR has been used to probe the diffusion and dynamics of IDRs in the condensed phase. Biophysical characterizations of IDRs in condensates have often adopted a minimal model where the samples contain only the protein region that is the main driver of LLPS. In some studies, other molecules such as nucleic acids that interact with the LLPS driver and participate in the condensate formation are also included, but the number of types of biomolecules in an NMR sample is typically minimal, while the cellular condensates contain a much larger number of different biomolecules.^43^ Furthermore, as in most protein NMR studies, the samples are often a truncated protein construct that contain the critical region that is capable of undergoing phase separation, and the working concentration required is hundreds of micromolar or higher. In some cases, crowding agents such as dextran and PEG are added to the sample to promote phase separation.^44,45^ NMR characterization of phase-separating proteins often requires samples of both disperse and condensed phases, and therefore, proteins with a low saturation concentration for phase separation are less ideal because the signal-to-noise ratio would be too low for the dispersed phase sample. To perform NMR measurements on the condensed phase of a phase separated protein sample, the droplets often fuse and settle down to the bottom of the NMR tube by gravity or centrifugation. This process often demands a large quantify of proteins, and such mass production can be challenging for many systems.

NMR diffusometry has been used to measure the translational diffusion of the germ-granule protein Ddx4^46^ and the diffusion of various small-molecule probes within the droplet.^46–48^ Pulse-field gradient NMR experiments, in which faster diffusion is indicated by greater signal loss, were performed by Kay and coworkers for Ddx4 and small-molecule probes with hydrodynamic radii ranging from 1.1 Å to 4.6 Å in the dispersed and condensed phases to measure their diffusion coefficients.^46^ Ddx4 diffuses in the condensed phase with a diffusion coefficient of 7.5 ± 0.4 × 10^−9^ cm^2^/s, 100 times slower that in the dispersed phase, while small molecules only diffuse 1.5 to 8 times slower, with the ratio increasing monotonically with their hydrodynamic radii. These studies together show that the impact of phase separation on diffusion is dependent on the size of the probe molecule,^46–48^ of which the translational diffusion rate decreases as the hydrodynamic radius increases.

NMR has also been used to address the driving force behind the self-assembly of proteins into condensates. Chemical shift perturbation is the more straightforward way to look for residues critical for LLPS, as chemical shifts are sensitive to environmental changes and report on molecular interactions. Fawzi and coworkers used chemical shift perturbation from ^1^H-^15^N HSQC spectra as a function of protein concentration to identify residues important for protein self-association.^49^ A local region with ∼20 residues that undergo larger chemical shift perturbation upon increasing concentration was identified for the C-terminal domain of the RNA-binding protein TDP-43, and mutations in this region disrupt phase separation. Although the self-assembly of the C-terminal domain of TDP-43 is driven by a localized group of residues, in most cases chemical shift perturbations for phase-separating proteins at increasing concentrations are observed across the entire protein region. In addition, while chemical shift perturbation can report changes due to interactions, they do not offer information of direct contacts. For probing contacts between residues within the condensed phase, nuclear Overhauser effect (NOE) was utilized to detect short-range (<6 Å) interactions via through-space dipolar couplings of ^1^H. To probe the intermolecular contacts within the condensates, a combination of differential isotopic labeling and heteronuclear filtering/editing is often carried out. Kay and coworkers carried out ^13^C-filtered, ^13^C-edited NOESY experiments on 10% ^13^C-labeled Ddx4 with 90% unlabeled Ddx4 to detect intermolecular contacts between labeled and unlabeled molecules.^46^ Among all amino acid types, Phe and Arg are most frequently found in intermolecular interactions detected by NOESY, supporting the importance of π-π and cation-π interactions in phase separation. Fawzi and coworkers performed ^13^C-HSQC-NOESY-^15^N-HSQC experiments on 50% ^13^C-lebeled and 50% ^15^N-labeled protein to detect intermolecular NOE from carbon-attached proton to backbone amide.^50,51^ The results showed highest NOE for Tyr and Gln and significant NOE signals for hydrophobic amino acids in the condensed phase of the prion-like-domain of enhanced filamentous growth protein 1 (Efg1),^51^ and showed that the condensed phase of the low-complexity domain of FUS is stabilized by hydrophobic, hydrogen bonding, and sp2-π interaction.^50^ NOESY experiments have also been used to identify contacts between two different IDRs that form co-condensates.^52^ Direct detection of intermolecular contacts by NOE expands the understanding of the chemistry that stabilizes the condensate formation from the original sticker-and-spacer model, where aromatic residues interact with each other as stickers to promote LLPS.^53^ Current NOESY-based work suggests that the intermolecular network in the condensed phase is not limited to interactions between aromatic residues, but also supported by a diverse set of interactions including aromatic-backbone, hydrophobic, charged, polar, and hydrogen-bonding.^46,50–52,54^

Dynamics of IDRs inside the condensate can be impacted by both macromolecular crowding and intermolecular interactions. NMR spin relaxation has been a major method to investigate the impact of phase separation on the motions of IDRs on the timescale of picosecond to nanosecond. ^15^N spin relaxation rates depend on the reorientation of ^15^N-^1^H bond vectors. The backbone flexibility of IDRs in the dispersed and condensed phases has been characterized by the measurement of the longitudinal (R_1_) and transverse (R_2_) relaxation rates and the heteronuclear ^1^H-^15^N NOE. Most phase-separating IDRs, including the proteasomal shuttle factor UBQLN2,^55^ the RNA-binding protein hnRNPA2,^56^ FUS,^52,57^ Ddx4,^46^ and elastin-like polypeptides (ELPs),^58^ have been reported to have increased R_2_ and heteronuclear NOE in the condensed phase, reflecting slowed down dynamics. Interestingly, the impact of LLPS on R_2_ seems to be larger than in NOE,^55,57^ implying a larger effect of phase separation on the segmental chain dynamics than on higher-frequency local motions. To quantify the effects of LLPS on the conformational dynamics at different timescales, Blackledge and coworkers conducted a detailed ^15^N relaxation analysis for the intrinsically disordered domain of the nucleoprotein of measles virus N_TAIL_ at different magnetic field strengths.^45^ By measuring R_1_, R_2_, heteronuclear NOE, and chemical shift anisotropy cross-relaxation η_xy_, and carrying out model-free analysis using three exponential components, they described the molecular motions of N_TAIL_ at different timescales that correspond to librational (∼50 ps), backbone dihedral angle sampling (ns), and segmental motions (∼10 ns).^59,60^ This analysis found that the correlation times of all three components are slowed down by 1.5-fold to fourfold in the condensed phase, and the amplitude of the fast component is substantially more restricted by LLPS, contrasting the expectation of a larger effect on the slower segmental motions from interpreting R_1_, R_2_, and heteronuclear NOE alone.

NMR relaxation dispersion has been powerful to quantify the interconversions between a highly populated state (ground state) and a sparsely population state (excited state) on the timescale of microsecond to millisecond. Using R_1ρ_ relaxation dispersion, Kay and coworkers probed conformational exchanges of Ddx4 within the condensates.^61^ The data suggest a model in which Ddx4 in the condensed phase exchanges between a ground state and a significantly populated excited state (30%) on the millisecond timescale. The excited state is characterized by an increased R_2_ value, consistent with increased molecular interactions, and no significant chemical shift differences from the ground state, which may be due to the weak and transient nature of the interactions. Similar dispersion profiles of slow exchange with a highly populated excited state (25%) were also observed for a non-phase-separating Ddx4 mutant at high concentration (370 mg/mL). The relationship between the observed conformational exchange and the formation and breaking of molecular network for LLPS remains to be explored.

### C. Non-stoichiometric clusters in subsaturated solutions

The formation of biomolecular condensates is described by the model of liquid-liquid phase separation (LLPS),^32,62^ where a sharp transition from 1-phase to 2-phase occurs beyond a saturation concentration. In the conceptual model of LLPS, the dilute phase is composed of monomers while the condensed phase is characterized by a higher protein density, stabilized through a network of intermolecular interactions. This model is challenged by recent findings of a distribution of protein clusters of different sizes below the saturation concentration.^32^ Using dynamic light scattering, fluorescence anisotropy, nanoparticle tracking analysis, microfluidic confocal microscopy, and transmission electron microscopy, Kar *et al.* characterized the heterogeneous distribution of clusters of phase-separating RNA-binding proteins. These clusters, with sizes ranging from 7 nm to 100 nm, shift toward to larger sizes as protein concentration increases, suggesting their role as the precursor of LLPS. The majority of the clusters contains tens to hundreds of proteins. Similar observation of non-stoichiometric clusters with ∼100 nm sizes has been observed in cells for RNA polymerase II,^38^ mediator,^33^ and transcription factors^63^ using super-resolution microscopy. NMR could be useful for characterizing biophysical properties of these clusters, such as protein conformational dynamics and rates of molecular exchange. Single-cell and single-molecule imaging of live cells by Chong *et al.* shows that clusters formed by transcription factors have optimal sizes for activity, while pushing the clusters toward larger LLPS droplets represses transcription.^64^ The functional roles of non-stoichiometric assemblies in subsaturated and phase-separated solutions, and the relationship between the size of the assemblies and their functions remain to be explored.^65^

## III. DYNAMIC INTERACTION MODES ORIGINATED FROM CONFORMATIONAL FLEXIBILITY AND MULTIVALENCY

**FIG. 2.**
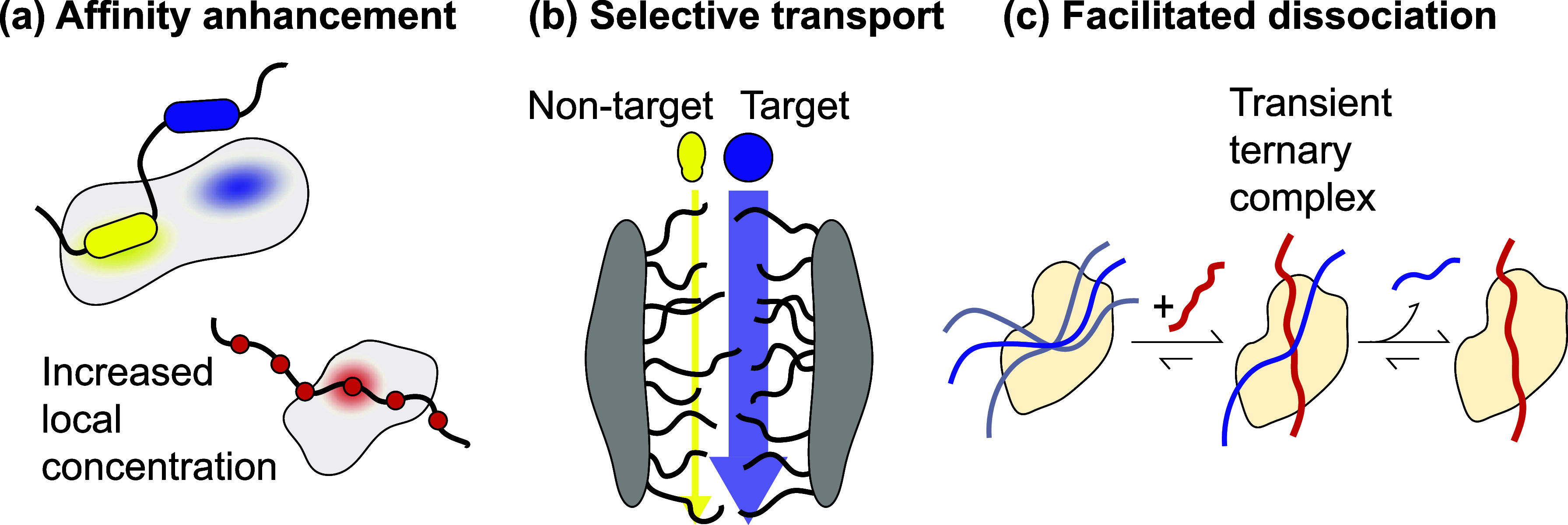
Dynamic interaction modes originating from conformational flexibility and multivalency. (a) Accumulation of weak interactions to enhance affinity. IDRs can form dynamic complexes through multiple weak interactions, enhancing overall binding affinity while maintaining conformational flexibility to respond to environmental changes. Examples include the viral protein E1A^66^ where bivalent interactions with two binding motifs connected via a linker (top) and pSic1^67^ where multiple binding motifs dynamically interacting with one binding site (bottom), both of which enhance overall affinity through an increase in local concentration. (b) Rapid exchange for selective molecular transport. IDRs can facilitate rapid exchange between binding partners to filter out nonspecific binding proteins, which is crucial for processes such as cargo transport through the nuclear pore complex. (c) Facilitated dissociation via transient ternary complexes. The flexibility of IDR complexes enables the invasion of binding competitors to form a transient ternary complex, driving faster dissociation compared to spontaneous dissociation.

Conformational flexibility and multivalency not only allow IDRs to form complexes and assemblies at various scales, together, they also open doors for dynamic interaction modes that facilitate functions not seen in the static view of molecular interactions. By participating in multivalent interactions while remaining dynamic (constantly changing conformations and alternating between free and partially bound states), IDRs introduce new interaction mechanisms that facilitate various regulatory functions (Fig. 2). The dynamic multivalency allows IDRs to achieve complex and responsive regulation that static proteins cannot.

### A. Increased affinity while being dynamic and responsive

Multivalency allows accumulation of multiple weak interactions to achieve stronger binding. The presence of multiple binding sites creates a high local concentration, and therefore, increases the chance of rebinding after dissociation. This kind of increased affinity has been widely observed in IDR systems.

A classic example is the work of Forman-Kay and coworkers on the yeast cyclin-dependent kinase inhibitor Sic1,^67^ which contains multiple phosphorylated serines/threonines that are recognized by its binding partner Cdc4. Binding of unphosphorylated Sic1 to Cdc4 is too weak to be detected by the intrinsic Trp fluorescence assay, while phosphorylated Sic1 (pSic1) with six phosphorylated residues binds to Cdc4 with a K_D_ of 0.6 *μ*M. Binding to the 80 kD Cdc4 is expected to lead to NMR line broadening for pSic1, yet the ^1^H-^15^N NMR correlation spectrum of ^15^N labeled Cdc4-bound pSic1 (99% bound) still display sharp resonances, suggesting that pSic1 does not form a stable, rigid complex with Cdc4. The pSic1-Cdc4 interaction was further probed with transferred cross-relaxation NMR experiments where the aliphatic protons of Cdc4 are saturated and the magnetization was transferred to the deuterated pSic1. Despite the large excess of pSic1 (molar ratio pSic1:Cdc4 = 26:1), significant transferred cross-relaxation for five out of six phosphorylated sites were observed in pSic1. These NMR observations suggest that when pSic1 binds to Cdc4, it is in a dynamic equilibrium with multiple sites exchanging on and off of the Cdc4 to achieve a higher overall affinity, potentially leading to ultrasensitivity in the cell.^68^

The enhanced affinity is often mediated by the linker connecting the interaction units, as exemplified in the study by Chemes and coworker on the bivalent binding of the intrinsically disordered adenovirus early region 1A (E1A) to human retinoblastoma (Rb) tumor suppressor.^66^ E1A binds to Rb with its two short linear motifs, each with a binding affinity of hundreds nanomolar. The two motifs are connected by a 71-residue disordered linker, and such tethering leads to a 4000-fold increase in binding affinity. Binding to Rb led to complete loss of NMR signals for the two motifs of E1A in ^1^H, ^15^N-TROSY spectra, but only modest intensity decreases for the linker. Affinity measurements with isothermal titration calorimetry (ITC) and fluorescence anisotropy experiments on single E1A motifs with and without the linker also further support the conclusion that the linker itself does not contribute to the stability of the complex. ITC measurements on the binding affinity of a single E1A motif to Rb bound to a separate second motif also showed no allosteric coupling between the two binding sites in Rb. Therefore, it was concluded that the linker enhances the affinity by increasing the effective concentration through tethering, and not because of additional linker interactions or allostery. It has been recognized that linkers can mediate effective concentrations through their length, sequence, and architecture.^69–71^ What Chemes and coworkers found in E1A is that although the linker length varies substantially among different viruses, their hydrodynamic radii remain constant, and the sequence and length of the linker might coevolve to conserve the dimension of the linker for optimal binding affinity.^66^

In addition to mediating binding involving well-defined interaction motifs with complementary binding interfaces, multivalency also manifests in a highly dynamic way without defined binding sites or interactions between specific residues. Using single-molecule FRET, Schuler and coworkers showed that the linker histone H1.0 (H1) and the histone chaperone prothymosin-α (ProTα), two intrinsically disordered protein with high opposite net charge, can form an ultra-tight complex with a picomolar affinity.^22^ Formation of the complex lead to small NMR chemical shift perturbation while the dispersion of ^1^H chemical shifts remain low in ^1^H-^15^N HSQC spectra, indicating disorder in the complex. No stable or transient secondary structures were detected by the C_α_ chemical shifts for H1 or ProTα associated with complex formation. Hydrodynamic radii of individual proteins and the complex measured by pulse-field gradient NMR and two-focus fluorescence correlation spectroscopy show the formation of one-to-one complex and the absence of larger scale oligomers and clusters. The longitudinal (T_1_) and transverse (T_2_) relaxation time of the ^15^N amide backbone measured by NMR show fast dynamics (ps – ns) for ProTα in both free and bound states, and the reconfiguration time (τ_r_) measured by single-molecule FRET with nanosecond fluorescence correlation spectroscopy for free proteins and the complex are in the range of 20–200 ns, consistent with that for disordered and unfolded proteins. While remaining disordered, increase in T_1_/T_2_ and τ_r_ were observed for the complex, indicating slowed down dynamics due to molecular interactions. One of the advantages of retaining the conformational flexibility in the ultratight complex is allowing the protein to respond to environmental changes such as presence of a new binding partner or changes in protein concentrations. For H1 and ProTα, there is rapid exchange of bound and unbound proteins at high concentrations that cannot be explained with a two-state model, where the complex dissociates before the rebinding.^24^ Instead, the dynamics of the H1:ProTα allows the formation of a transient ternary complex where a new molecule binds to the exposed sites and eventually becomes fully bound to replace the former molecule. The dynamic nature of disordered complexes also allows accelerated dissociation of H1 from the nucleosome by ProTα, which is discussed in Sec. III C.

The conformational freedom of IDRs allows them to strengthen the binding via multivalent interactions while remaining dynamic and accessible for additional binding or post-translational modification. Such flexibility allows the tight binding observed in IDRs to be reversible and responsive to changes.

### B. Rapid exchange for selective molecular transport

Multivalency does not always lead to large enhancement in binding affinity, and this can also be functionally advantageous. In the case of FG nucleoporins (FG Nups), which form the inner channel of the nuclear pore complex, Cowburn and coworkers found that divalent interaction only increases the affinity by twofold compared to monovalent interaction.^25^ Using NMR titration, they measured binding affinities of a series of FG Nups containing different numbers of FG motifs binding to the transport factor NTF2, and found only modest increase in the binding affinity as the valency increases, and no significant increase beyond having 6 motifs. TROSY-HSQC spectra of NTF2 with titration of FG Nups containing 1, 3, and 6 motifs show the same set of affected residues shifting linearly, indicating the same binding mode for all FG Nups constructs regardless of the valency and fast exchange rates for binding. ITC further shows almost perfect offset of an increasing ΔH by an increasing -TΔS as the valency increases. Dynamic light scattering indicates a 1:1 complex at high concentrations. Together, these results suggest a model in which only a small fraction of the FG motifs is occupied and there is rapid exchange of transport factors among FG motifs. Even with excess of transport factors, the FG motifs do not get saturated because the enthalpy gained from being fully bound does not compensate for the entropy cost of restricting the conformational freedom in the bound state. As the result of the enthalpy-entropy balance, the avidity between FG Nup and transport factor is low, and increasing in local concentrations lead to slight increase in the overall affinity. An FG Nup typically contains 5 to 50 motifs that bind to transport factors carrying specific cargoes. The rapid exchange of transport factors among and array of FG motifs can be advantageous for cargo transport, where the frequent contact enhances the selectivity as nonspecific interactions are diminished. The retention of conformational flexibility of FG Nups as a result of entropy-enthalpy compensation prevents the formation of a tightly bound complex that does not release the cargo.

### C. Facilitated dissociation through transient ternary complex

The dynamic multivalent interactions of IDRs not only allow rapid association and dissociation of the two molecules in the complex, they also provide a way to initiate the dissociation by a different protein via the formation of a transient ternary complex. Sometimes referred to as facilitated dissociation^72^ or molecular stripping,^73^ this active dissociation mechanism has been reported for protein systems with a variety of degrees of disorder, from short linkers in multidomain proteins to complete disordered protein complexes.

Facilitated dissociation was first demonstrated in the RelA-p50 heterodimer of NF-ĸB family of transcription factors. Both the RelA and p50 monomers contain a dimerization domain and a N-terminal DNA binding domain (NTD) connected by a 10-residue linker. RelA-p50 (referred to as NF-ĸB here) binds to the DNA primarily through the two NTDs. The negative regulators IĸBα facilitates the dissociation of NF-ĸB from the DNA by forming a transient ternary complex, as captured in stopped-flow fluorescence kinetic experiments^74^ and NMR.^75^ The formation of the ternary complex is enabled by the relative interdomain motions of the NTDs, leading to a conformation of NF-ĸB that is partially bound to the DNA for IĸBα to invade the binding cavity and eventually replace DNA.^76^ The multivalent interaction and the conformational flexibility of NF-ĸB in complex of the DNA enabled by the disordered linkers is the key to molecular stripping by IĸBα. Binding to IĸBα rigidifies the relative motions of the NF-ĸB NTDs, and therefore, lock NF-ĸB in a conformation that cannot rebind to the DNA.^76^

Such unidirectional inhibition is also found in the competition between the transcription factor HIF-1α and its negative regulator CITED2 for the binding to the general transcriptional coactivator CBP.^21^ Both HIF-1α and CITED2 are intrinsically disordered proteins containing helical motifs and bind to the TAZ1 domain of CBP with similar affinity, but when TAZ1 is mixed with equal molar of HIF-1α and CITED2, only the binary complex CITED2:TAZ1 is observed by NMR. This unidirectional competition is due to active replacement of HIF-1α from TAZ1 by CITED2. Stopped-flow fluorescence experiments show that the dissociation of HIF-1α from the complex it forms with TAZ1 is accelerated by CITED2 and the rate is proportional to the concentration of CITED2, indicating the formation of a transient ternary complex. Both HIF-1α and CITED2 contain a αA helix that compete for the same binding site on TAZ1, but the HIF-1α αA helix is flexible in the binary complex, as indicated by the low ^1^H-^15^N NOE, while the CITED2 αA helix in the binary complex is rigid and display high ^1^H-^15^N NOE. The conformational flexibility in the αA binding surface of the HIF-1α:TAZ1 complex allows the CITED2 αA to invade the partially engaged site and eventually replace HIF-1α. NMR titration experiments and line shape analysis on truncated and mutated CITED2 constructs added to HIF-1α-bound ^15^N TAZ1 capture the ternary complex and show that the unidirectional interaction can be described with a 3-state model: rapid binding of the αA helix of CITED2 to TAZ1 followed by the binding of the other two motifs in CITED2 to completely replace HIF-1α.^77^

A third example of facilitated dissociation is the release of the histone linker H1.0 (H1) from the nucleosome driven by the polyelectrolyte competition between the histone chaperone prothymosin-α (ProTα) and the nucleosomal DNA. In Sec. III A, we mentioned that H1 and ProTα form a dynamic, disordered complex with picomolar affinity and form a transient ternary complex at high concentrations leading to rapid exchange of molecules in the binary complex. Using single-molecule FRET, Heidarsson and coworkers further showed that ProTα enhances the dissociation of H1 form the nucleosome by 2 orders of magnitude via the formation of a transient ternary complex of H1.0:nucleosome:ProTα, providing an explanation for the discrepancy between the 3-h *in vitro* residence time for H1 on the nucleosome and the 1-minute residence time in cells.^23^ The highly positively charged H1 forms a dynamic complex with the negatively charged nucleosome DNA via multivalent interactions, where H1 monomers constantly engage and disengage with the DNA. The conformational fluctuation within the H1:nucleosome complex allows ProTα to invade the partially unbound H1 and form a transient ternary complex, during which contacts between H1 and ProTα increase and contacts between H1.0 and DNA decrease. Eventually, ProTα completely replaces the DNA and forms a binary complex with H1.

The three examples show how switch-like dissociation of tight complexes is enabled by the combination of multivalency and conformational flexibility through various degree of intrinsic disorder from short linkers to IDR with secondary structures to completely structureless IDRs.

## IV. NARROWING THE GAP BETWEEN MOLECULAR DESCRIPTIONS AND CELLULAR FUNCTIONS OF IDRS

Current NMR studies on IDRs are largely based on the reductionist approach where proteins of interest are truncated to sizes that can be handled by NMR and studied in buffers in test tubes. These studies have provided valuable insights, particularly on the discovery and quantitative characterization of structural properties and binding interactions of IDRs, including novel interaction modes discussed above. However, a more complete understanding of IDRs requires biophysical characterization of the full-length proteins, large protein complexes, and in the context of cellular environment. Below we discuss emerging NMR methods that can narrow the gap between current *in vitro* molecular descriptions and cellular functions of IDRs.

### A. Tackling large proteins in different phases with solid-state NMR

Moving toward a more complete molecular understanding of full-length proteins with IDRs and their complexes, while solution NMR can struggle to detect large and slow tumbling proteins, but solid-state NMR with magic-angle spinning (MAS) is not limited by molecular size and can observe both a variety of phases and dynamics a protein may assume.^78^ In MAS NMR, polarization transfer methods can be used to selectively retain signals associated with regions of the protein engaging in either rigid, intermediate, or liquid-like dynamics: dipolar cross-polarization (CP) methods are used to monitor rigid regions; direct polarization methods to capture intermediate regions; and scalar-coupling based (J-based) INEPT methods are used to monitor mobile regions.^78–82^ The power of such dynamic editing methods was demonstrated by Zhou and coworkers on the fuzzy membrane association with the protein ChiZ, a part of the divisome complex in *M. tuberculosis.* Line broadening observed upon ChiZ binding to liposomes mimicking the plasma membrane renders solution NMR impossible for identification of the ChiZ binding region. INEPT-based MAS NMR indicates that most of the protein remains dynamic upon binding and complementarily CP-based NMR allowed for the identification of residues implicated in binding.

Hypotheses generated from solution NMR using short constructs which isolate functional regions can be examined in a more complete biomolecular context using solid-state MAS NMR. Histones and chromatin-associated proteins are one such example where juxtaposing information from liquid and solid-state NMR is especially useful to interrogate biomolecular behavior. Foundationally, MAS NMR has been used to observe the structure and dynamics of histones in nucleosome core particles, nucleosome arrays, and in a highly compacted heterochromatin-like state; MAS NMR contributions to elucidating chromatin structure and dynamics and current technical guidance have been recently reviewed.^82–84^ Solution NMR spectroscopy used to monitor binding of HP1α chromoshadow domain (CSD) to a truncated histone H3 construct (residues 1–59, encompassing the disordered tail and a small core region) indicated the CSD contacts a PXVXL motif in the core region in isolation.^85^ In the context of an intact nucleosome, the core is invisible to solution-state measurement, whereas both the dynamic tails and rigid core can be observed using dynamic editing MAS NMR techniques. No discernable changes to the nucleosome core were observed when probing the interaction in the context of intact nucleosomes, supporting the idea that HP1α interacts with nucleosomes primarily through chromodomain and methylated histone H3 tail contacts.^86^

MAS NMR has also been powerful for probing fibril structure and mechanisms contributing to protection from aberrant fibrillization, informing pathogenicity, as demonstrated for TDP-43,^87^ FUS,^88–90^ and TIA.^91^ As previously mentioned, Fawzi and coworkers identified a region of TDP-43 important for protein association and oligomerization using solution NMR. An α-helical region which contacts a C-terminal β-strand region was shown both to be important for phase separation of TDP-43.^49^ However, solution NMR was unable to probe secondary structure of these regions or their contacts in the hydrogel state. Conversely, MAS NMR of aged liquid droplets in the hydrogel state reveals the existence of a second aggregation core formed after a period of days and encompassing the β-strand region and supports a mechanism where the β-strand region contributes to preventing pathogenic fibril formation.^87^ For the low complexity domain of FUS (FUS-LCD), a structural model of fibrils generated for solid-state NMR revealed an ordered fibril core flanked by disordered segments, with evidence of hydrogen bonding between specific residues in the fibril core.^89,90^ Although distal to the fibril core, mutation of glycine to glutamate in FUS-LCD (G156E) increases patient risk for ALS and drives both LLPS and FUS fibrillation,^92^ a surprising phenomenon as the addition of negative charge through serine/threonine phosphorylation throughout the FUS-LCD was found to be protective to LLPS and fibrillation.^89,90^ Dynamically edited techniques permit study of FUS-LCD G156E, as it is present in a region shown to remain mobile in previous structural work but accelerates LLPS and fibrillization, which causes affected regions in the fibril core to become rigid. INEPT-based experiments conducted on aging samples over a period of weeks revealed that the dynamic regions of FUS-LCD G156E underwent significant losses in overall signal intensity in the first week, whereas the total signal intensity from dynamic regions of wild-type FUS-LCD remained relatively constant over a three-week period. Differences in one set of glycine resonances in FUS-LCD G156E lends further evidence to the possibility of inherently different dynamic environments between wild-type and mutant FUS-LCD. These dynamic differences could influence inter-protein interactions accessible to the FUS-LCD, including contributors to amyloid fibrils such as steric zippers.^88^ A recent preprint on the conformational distribution of the frozen solution of phase separated FUS-LCD also highlights the importance of solid-state NMR in understanding the structural basis of phase transition and fibril formation of IDPs.^93^

Dynamic nuclear polarization (DNP)-enhanced MAS NMR can be used to alleviate drawbacks to MAS NMR resulting from low signal-to-noise ratio inherent to the technique. The low signal-to-noise ratio contributes to the major limitations of MAS NMR for the study of biomolecular complexes: sample quantity and necessity of sample stability over prolonged periods. In summary, DNP-enhanced MAS NMR utilizes paramagnetic agents as a source for polarization transfer from electron spins to nuclear spins to enhance signal-to-noise. Its principles and broader applications have recently been reviewed in detail.^94,95^ Debelouchina and coworkers have recently discovered the ability of DNP-enhanced MAS NMR to report on aromatic residues previously invisible to MAS NMR in nucleosome arrays, a particularly difficult sample to prepare at scale necessary for traditional MAS NMR.^96^ DNP-enhanced MAS NMR is also an emerging method to observe the conformational ensemble of IDRs. At the low temperature required for DNP (100 K), a multitude of sampled conformations will be captured. The combination of conformations results in broad peaks whose features can be used to assess sampled secondary structure through deviations from random coil behavior^97^ or torsion angle.^98^

### B. Exploring IDRs in native cellular environments with in-cell NMR

Ideally, proteins should be studied in their native environment—the cell. Over the past decades, progress on in-cell NMR has allowed atomic measurements of protein folding stability,^99,100^ protein–protein interactions,^101,102^ conformational dynamics,^103,104^ mobility,^105,106^ and even structure determination^107^ within living cells including *E. coli,*^99,100,105,106,108^ yeast,^109^ *X. laevis* oocyte,^110,111^ insect cells,^112^ and mammalian cells.^103^ Compared to folded proteins, which suffer from low NMR signal-to-noise ratio due to slower tumbling in cells, the flexibility of IDRs reduces the impact of the viscous cellular environment on their NMR spectra.^105,106^

Among IDRs, α-synuclein has been a major target for in-cell NMR and a model system for method development due to its relevance in Parkinson's disease and high-resolution in-cell NMR spectra.^103,105,106,108,113–117^ Previously, it has been hypothesized that while α-synuclein is intrinsically disordered, it adopts a helical tetrameric form in cells. In-cell NMR studies by Pielak and coworkers, where α-synuclein is overexpressed in *E. coli*, show that it remains disordered in the *E. coli* cytoplasm.^105,108^ Selenko and coworkers further introduced α-synuclein inside a number of mammalian cell types, including neuronal and non-neuronal, by electroporation and demonstrated that α-synuclein remains as intrinsically disordered monomers inside the native environment of cells, as indicated by the two-dimensional ^1^H-^15^N spectra.^103^ Furthermore, they found that unmodified α-synuclein becomes N-terminally acetylated inside the cells, shown as decreased resonance intensities for the first ten residues, suggesting that α-synuclein can be acetylated post-translationally to adopt its functional form, not only co-translationally. To investigate the dynamic properties of acetylated α-synuclein in cellular environments, Selenko and coworkers performed ^15^N NMR relaxation on α-synuclein in mammalian cells and *in vitro* with various crowding agents. From R_1_, R_2_, and ^1^H-^15^N NOE, they obtained residue-specific rotational correlation time τ_c_ on the nanosecond timescale and the exchange term R_ex_ for the microsecond to millisecond timescale. A universal increase in τ_c_ reflects the decrease in fast dynamics due to the viscous *in vitro* and cellular conditions, while nonuniform changes in the exchange terms were found for specific regions of α-synuclein including Tyr 39. Through varying *in vitro* salt concentrations and site-directed mutagenesis, they found that the exchange behavior of α-synuclein is influenced by electrostatic and hydrophobic interactions. Using NMR paramagnetic relaxation enhancement (PRE) and electron paramagnetic resonance (EPR), they also found out α-synuclein adopts a more compact conformation in cells than the in buffer, with the aggregation-prone residues shielded from the cytosol. A more recent in-cell NMR study by Hiller and coworkers demonstrated various chaperone interactions with specific regions of α-synuclein including Tyr 39 in mammalian cells.^115^ ^19^F in-cell NMR has also been used to monitor conformational changes and membrane insertion of α-synuclein into mammalian cells through cell-penetrating peptides.^117^ Diffusion of α-synuclein within bacterial cells has been measured with pulse-field gradient NMR, which can also be applied as a spectral editing tool to eliminate extracellular species.^116^ Other IDRs that has been studied with in-cell NMR include prokaryotic proteins FlgM^118^ and Pup^119^ in *E. coli* cells, FG Nups in *E. coli* and yeast,^104,109^ and Tau in *X. laevis* oocytes.^110^

In-cell NMR has advanced significantly due to methodological improvements. Given the lower signal-to-noise ratio of in-cell NMR spectra and the inherent limited cell lifetime, enhancing the signal-to-noise per unit time is essential. Techniques ^1^H-^15^N SOFAST-HMQC^120^ and BEST-HSQC^121^ have been widely used for increasing the sensitivity of in-cell NMR. To extend the cell life, bioreactors for keeping cells alive in NMR spectrometers have been developed.^122–124^ Furthermore, recent development in dynamic nuclear polarization (DNP) solid-state NMR shows the potential of detection of micromolar cellular concentrations in frozen cells,^125^ which aligns with typical physiological protein concentrations. Ongoing developments in in-cell NMR, including cellular lifetime prolongation with bioreactor devices,^122–124^ NMR sensitivity enhancement with advanced polarization methods at higher temperature (>200 K),^126^ and spectral resolution improvement with ^13^C direct-detect NMR,^127^ are expected to provide a more comprehensive understanding of IDR structure and dynamics in the native cellular environment. Potential future directions include the characterization of in-cell IDR assemblies and condensates, as well as IDRs in the nucleus and other cellular compartments.

## V. CONCLUSIONS AND PERSPECTIVE

This review has highlighted recent conceptual breakthroughs in the study of IDRs and the important role of NMR in these studies, from the characterization of IDR assemblies at various scales to the kinetic/thermodynamic consequences and functional implications of dynamic IDR interactions. We envision that NMR will continue to be critical in understanding the biophysics and functions of IDRs. A future direction of studying IDR assemblies is to look at how mixtures of biomolecules, including IDRs and nucleic acids, are incorporated into clusters and condensates. Key questions include: How are diffusion and dynamics influenced by different components? What molecular contacts stabilize these various molecular networks within clusters and condensates? What are the molecular determinants behind the relationship between cluster size and function? NMR characterization of stoichiometric IDR interactions will continue to uncover dynamic modes that expand the functional repertoire of macromolecular interactions. With advancement in solid-state NMR and in-cell NMR, it is becoming possible to study full-length proteins in various phases and within cellular environment. These developments are narrowing the gap between *in vitro* molecular descriptions and the cellular functions of IDRs, providing a more comprehensive understanding of their roles in cellular processes.

## Data Availability

Data sharing is not applicable to this article as no new data were created or analyzed in this study.
